# Influence of two different printing methods on the accuracy of full-guided implant insertion – a laboratory study in undergraduate dental students

**DOI:** 10.1038/s41405-025-00295-y

**Published:** 2025-01-26

**Authors:** Matthias C. Schulz, Michael Krimmel, Christina Weismann, Pablo Kaucher-Fernandez, Bernd Lethaus, Nils Kristian Mann

**Affiliations:** 1 Department of Oral and Maxillofacial Surgery (Head: Prof. Dr. Dr. Bernd Lethaus), University Hospital Tübingen, Eberhard Karls Universität Tübingen, Osianderstr. 2-8, D-72076 Tübingen, Germany; 2 Department of Orthodontics (Head: Prof. Dr. Bernd Koos), University Hospital Tübingen, Eberhard Karls Universität Tübingen, Osianderstr. 2-8, D-72076 Tübingen, Germany; 3 Department of Prosthetic Dentistry (Head: Prof. Dr. Fabian Hüttig), University Hospital Tübingen, Eberhard Karls Universität Tübingen, Osianderstr. 2-8, D-72076 Tübingen, Germany

**Keywords:** Extended skills training in dentistry, Dental implants

## Abstract

**Objectives:**

The aim of the present study was to compare the accuracy of fully guided implant insertion in vitro achieved by two fabrication methods in a cohort of undergraduates. We hypothesized that both methods achieve a comparable accuracy.

**Methods:**

Surface scans and cone beam computed tomography images of 48 mandibular models were matched. For each model, two surgical guides enabling a fully guided implant insertion in the region of the first molar on the left or the right side were virtually designed. Fabrication by either Digital Light Processing (DLP) or Fused Filament Fabrication (FFF) followed. Subsequently, 96 implants using the guides were inserted into the models by 48 undergraduate students. The accuracy of the implant insertion was assessed radiographically, followed by statistical analysis. Additionally, all participants completed a questionnaire.

**Results:**

The implants inserted using guides made by DLP showed a higher accuracy compared to guides made by FFF. The mean three-dimensional deviation was 1.94 ± 1.05 vs. 3.35 ± 2.03 degrees (*p* < 0.001). The evaluation of the questionnaires revealed mainly theoretical knowledge and a pronounced interest in implant dentistry.

**Discussion:**

The main hypothesis has to be rejected as there were statistically significant differences in accuracy. However, it is possible to teach students the principles of guided implant dentistry and the digital workflow. Furthermore, the initial and running costs for the FFF workflow are substantially lower enabling a higher practicability for undergraduate education.

**Conclusion:**

Despite the lower accuracy of the templates made from FFF the method seems to be suitable for laboratory hands-on courses for undergraduates.

## Introduction

Implant dentistry has become an established therapy option over the recent decades. The ideal implant position regarding anatomical and prosthetic aspects has a crucial influence on the long-term success [1]. In order to achieve a high transfer accuracy of the planned implant position into the clinical situation, fully guided implant insertion has shown the lowest degree of deviation [2]. Currently, with the increased availability of cone beam computed tomography (CBCT), intraoral scanners and three-dimensional printers as well as decreasing financial effort, static computer-assisted implant surgery (s-CAIS) is established in the clinical practice [3, 4]. Currently, there is a widespread variety of applications of additive manufacturing (AM) in oral surgery and dentistry [5]. The methods are not only differing in accuracy and thus, in their fields of applications, but also considering their initial and running costs [6]. It could be shown that devices made from DLP printers are higher accurate compared to devices made by FFF printers [6, 7]. Thus, the application of devices made from DLP comprises individual impression trays, dental casts and surgical guides [6]. In contrast, devices made from FFF were not yet considered as suitable for the fabrication of surgical guides as they showed a lack of precision. However, there were notable differences in the initial and running costs of both printers in favor of the FFF printers [7].

In the course of the mentioned developments, there is an increased demand for an extended education of undergraduates in implant dentistry [8–11] Apart from theoretical knowledge, a practical training of the students would be desirable before treating patients [12]. In order to provide a practical experience for undergraduates, hands-on courses are an established method despite the financial effort for the organizing dental school [13]. This led to the search for a practicable and cost-effective approach to allow a hands-on course with individual surgical templates for each participant.

The aim of the present study was to evaluate the accuracy of fully guided implant insertion in mandibular models achieved with guides fabricated from Digital Light Processing and Fused Filament Fabrication in a cohort of undergraduate dental students. We hypothesized, that the accuracy of both methods is comparable. Furthermore, other individual factors potentially having an influence on the accuracy, e.g. age, gender, handedness and an education completed prior to studying dentistry were recorded. Additionally, the participants were asked to complete a questionnaire assessing their current knowledge in implant dentistry.

## Methods

### Ethics

Before the start of the study, the study protocol was reviewed and approved by the local Ethical Review Board (file reference: 123/2022BO2). The examination was conducted according to the Declaration of Helsinki in its current version [14]. All students participating in the study gave their written informed consent before taking part.

### Manufacturing of the surgical guides

For the study, 48 mandibular models (Implantec Dentallabor, Amstetten, Germany) with each a single-tooth gap in the region of the first molar on both sides were used. The models were numbered consecutively. First, a surface scan of each model was performed using a laboratory scanner (Ceramill Map 600, Amann Girrbach GmbH, Pforzheim, Germany). The data sets were saved as Standard Tessellation Language (STL). Next, a three-dimensional radiographical scan of each model using a cone beam computed tomography (CBCT, Veraviewepocs 3D, J. Morita Corporation, Osaka, Japan) was carried out. The following parameters were set-up: tube voltage: 90 kV; current: 5 mA; exposure time: 9,4 s field of view: 100 × 100 × 50 mm; voxel size: 0.125 × 0.125 × 0.125 mm. The radiographical data was saved in Digital Imaging and Communication in Medicine (DICOM) format. The CBCT data set and the surface scan were imported into the implant planning software (coDiagnostiX™ Version 10.2, Dental Wings, Chemnitz, Germany). Subsequently, the implant positions in regions 36 and 46 were planned according to prosthetic and surgical requirements by an experienced dental technician supervised by an experienced oral surgeon. A surgical guide was virtually designed for each planned implant position with fenestrations in region 31/41, 35/45, 37 and 47 in order to allow the fit check. The material thickness was set up at 3 mm for both materials and an off set of 0.1 mm for DLP printing (metal sleeve fitting: 0.0 mm) and 0.2 mm for FFF printing (metal sleeve fitting: 0.1 mm) was chosen. The surgical guides were saved as STL data. Next, one guide per model was printed using fused filament fabrication with a Prusa MK3S (Prusa Research a.s., Prague, Czech Republic) with acquisition costs of approximately 900 € and Clear Base Support by Arfona® (Arfona LLC, Brooklyn, USA) accounting approximately 250 € for 1000 g of the material. The other one was manufactured using digital light processing with a RapidShape D30II (Rapid Shape GmbH, Heimsheim, Germany) with acquisition costs of approximately 20.000 € and V-Print SG Resin by VOCO (VOCO GmbH, Cuxhaven, Germany) accounting approximately 285 € for 1000 g of the material. The distribution of the guides was as follows: for models with uneven numbers, the guide made by DLP was used for implant insertion in region 36 and the guide made by FFF in region 46. For models with even numbers, the manufacturing methods of the guides were applied vice versa: in region 36, the guide made by FFF was used for implant insertion and the guide made by DLP in region 46. After manufacturing the guides, a metal sleeve (T-sleeve, Ø 5 mm, H 5 mm, guided – stainless steel, Straumann Deutschland, Freiburg, Germany) was manually inserted. Finally, the guides were checked for their fit on the respective model.

### Hands-on course

**Fig. 1:**
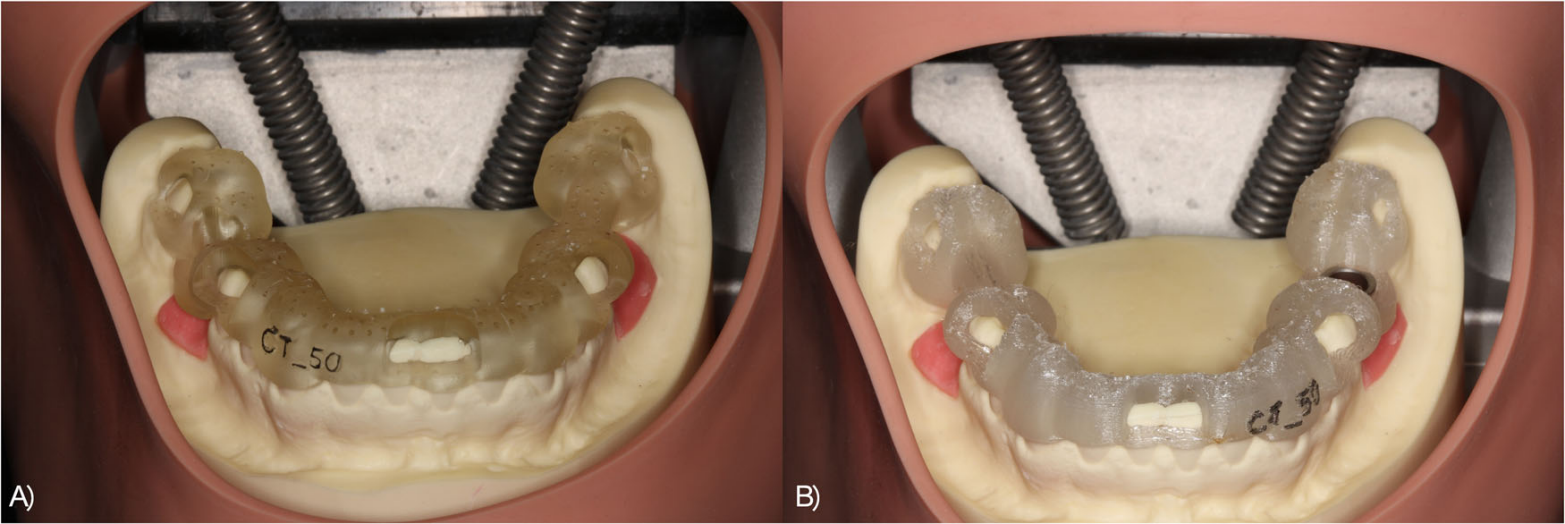
Guides positioned on the mandibular models in the phantom head. **A** Guide fabricated by Digital Light Processing for implant insertion in region 46. **B** Guide fabricated by Fused Filament Fabrication in region 36. The different surface structures are obvious.

All participants provided their consent prior to the study. First, the participants were asked to complete a form containing demographic data and an evaluation of existing knowledge considering implant dentistry. Following a theoretical introduction into the planning software and the implant system, the participants were asked to perform a virtual implant planning with the software and the implant insertion in the mandibular models using the guides. In order to resemble a patient situation, the models were mounted in phantom heads with a soft tissue mask (Phantomkopf PK2, Frasaco GmbH, Tettnang, Germany). The phantom situation is shown in Fig. 1. The participants assessed the fitting of both guides on the respective model according to a Likert scale (1–5, 1-good fitting – 5-poor fitting). Subsequently, the implant cavities were prepared according to the manufacturer’s protocol using the guided surgical tray (Straumann, Freiburg, Germany) and a surgical unit (Surgic-XT, NSK Europe, Eschborn, Germany). Two dummy implants (Bone Level RC 4.1 × 10 mm, Straumann, Freiburg, Germany) were inserted. The time required for the implant insertion from positioning the guides to the removal of the insertion abutment was recorded. The participants were asked to complete a questionnaire following the course to evaluate and suggest potential improvements. Furthermore, it was requested to state potential interests (implant planning, incision techniques, surgical methods, suture techniques, impression techniques and prosthetic options) of the participants for future hands-on courses with multiple options to choose.

### Evaluation

**Fig. 2:**
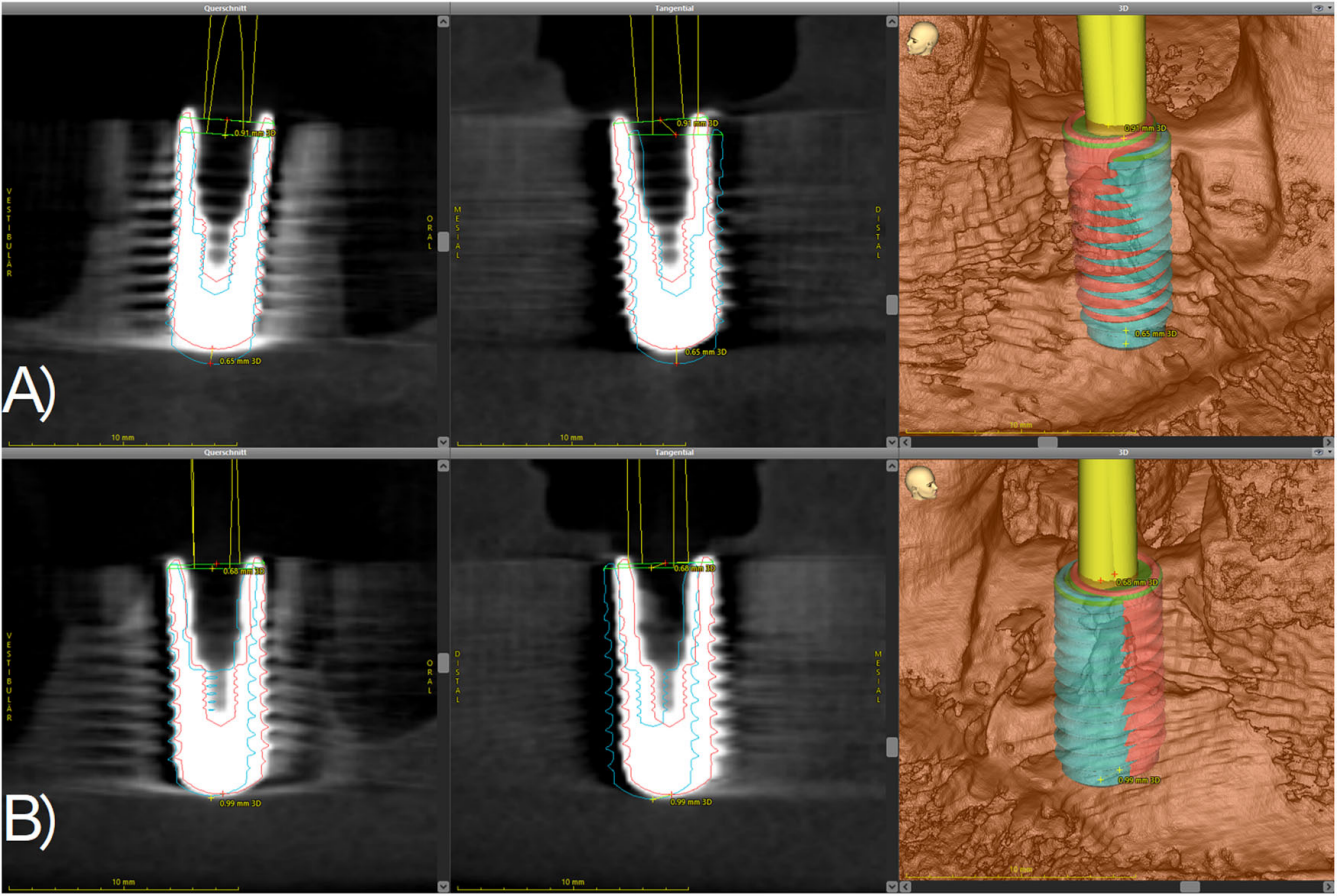
Scheme of the superimposition of the planned (blue) and the actually achieved (red) implant position. **A** The implant in region 36 (upper) shows a slightly mesial deviation from the planned position. Furthermore, the implant is placed a bit too crestal compared to the planned position. **B** The implant in region 46 (lower) shows a slight oral and mesial mismatch compared to the planned position.

**Fig. 3:**
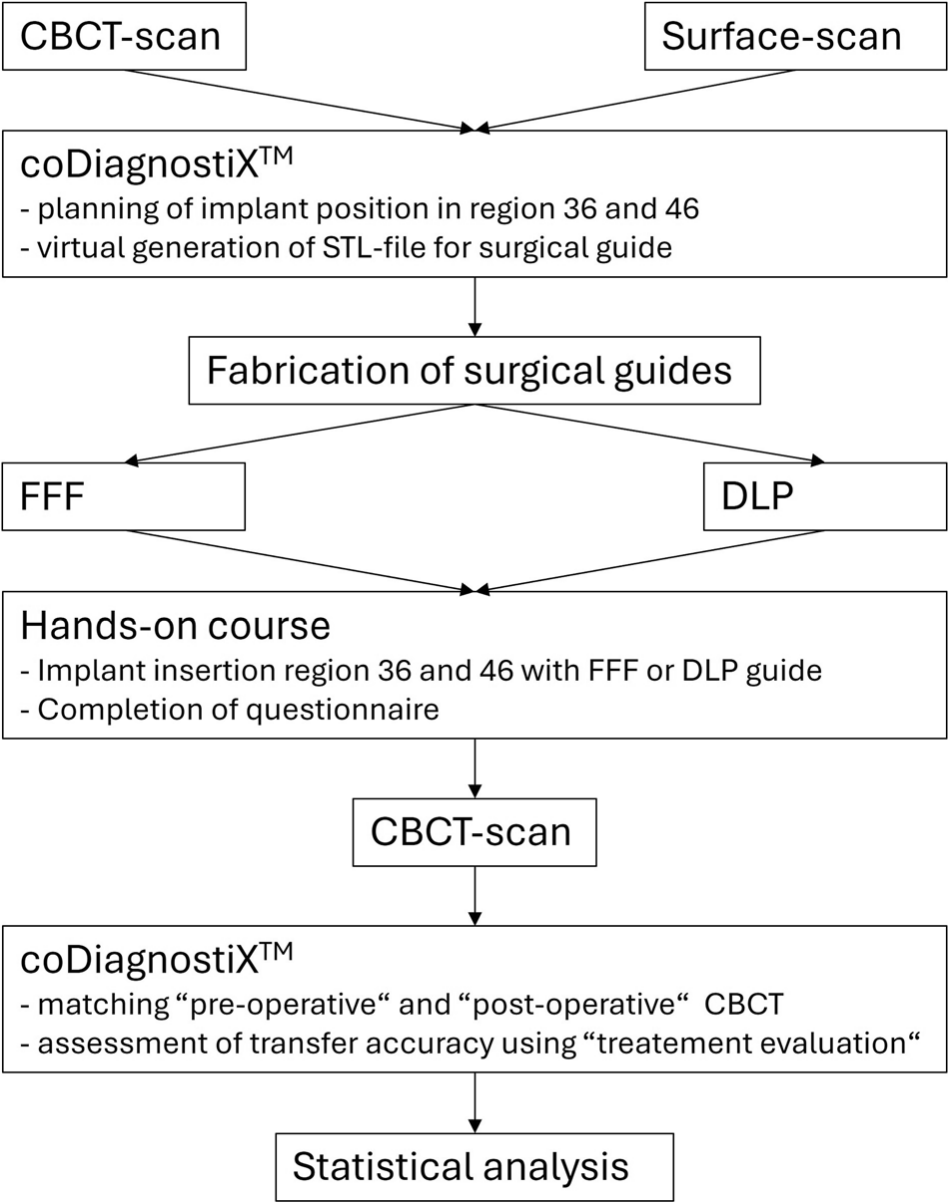
Flowchart of the study course. Depiction of the process from the initial Cone Beam Computed Tomography (CBCT) scan to the statistical analysis.

After the hands-on course, a CBCT of each model was performed using the same parameters as described above. The DICOM data sets were imported into the planning software. Using the “treatment evaluation” tool of the software, the virtual planned implant position and the CBCT scan of the manually inserted implants were superimposed as described previously [15, 16]. Next, the outline of the implants was congruently placed over the radiographical image of the achieved implant position (Fig. 2). After matching the data, the software calculated the horizontal, sagittal, vertical and three-dimensional mismatch at the implant base and tip and the three-dimensional deviation between the planned and implant positions achieved by the participants. In order to minimize matching errors, each measurement was performed three times. All values and the data obtained from the questionnaires were recorded in an MS Excel® chart (Microsoft Inc., Redmond, Washington, USA). The exemplary flow of the study is depicted in Fig. 3.

### Statistical analysis

The statistical analysis was performed in cooperation with the Noack Statistik GmbH, Bonn, Germany, using SPSS Statistics (version 28.0.1.1, IBM Corporation, Armonk, New York, USA). The mean values and their standard deviations were calculated. In order to test whether there is a normal contribution of the variables, a Shapiro–Wilk test was carried out. For the comparison of the effects of the different variables, a multivariate regression was performed. For the comparison of the fields of interests between genders the χ² test was utilized. The level of statistical significance was set up at α = 0.05.

## Results

### Demographic data

A total of 48 students (37 female, 11 male) took part in the course. The mean age was 26.30 ± 3.42 years ranging from 21.1 to 36.8 years. The distribution of handedness revealed 43 right-handed and 4 left-handed participants. One participant did not state the handedness. A majority of 27 students had completed an education prior to studying dentistry. The detailed demographic data is shown in Table 1.

**Table 1:** Demographical data of the participants

	Group	Dental students
Parameter		
Age	<25 years	19
	>25 years	29
Gender	Female	37
	Male	11
Handedness	Left	4
	Right	43
	Not stated	1
Professional training before dental school	No	20
	Not stated	1
	Yes	27
	Dental assistent	13
	Dental technician	8
	Other	6

### Differences between virtually planned and really inserted implant

When analyzing the values, no normal distribution was found. Based on the central limit theorem, that did not affect the results. Based on the assessment of the participants, the fitting of the guide made by DLP was rated with 1.37 ± 0.47 corresponding to a good fitting compared to 2.69 ± 1.13 corresponding to a satisfactory fitting for the guide made by FFF. This difference was statistically significant (*p* < 0.01).

As the accuracy measurements were carried out three times for every parameter, a total of 288 measurements was included into the analysis. Except for the vertical mismatch, the mismatch at the implant tip was higher compared to the implant base. The differences between virtually planned and actually achieved implant positions were lower for the implants inserted by using guides made by DLP compared to guides made by FFF. For the three-dimensional angle, the three-dimensional mismatch at the implant base and tip and the mesio-distal mismatch at the implant base and tip the differences reached a statistical significance. The detailed data is shown in Table 2.

**Table 2:** Mean values and their standard deviations (SD), 95% confidence interval (CI) and *p*-values regarding the different method of fabrication.

Parameter	Fabrication method	Mean value	±SD	Mean difference	Lower limit 95% CI	Upper limit 95% CI	*p*-value
Three-dimensional angulation in degrees	FFF	3.35	2.03	1.41	3.34	3.36	**<0.001**
	DLP	1.94	1.05		1.93	1.95	
Three-dimensional mismatch at implant base in mm	FFF	0.97	0.39	0.27	0.97	0.98	**<0.001**
	DLP	0.70	0.43		0.70	0.71	
Mesio-distal mismatch at implant base in mm	FFF	−0.30	0.38	−0.17	−0.30	−0.30	**<0.001**
	DLP	−0.13	0.29		−0.14	−0.13	
Bucco-lingual mismatch at implant base in mm	FFF	0.11	0.57	0.07	0.10	0.11	0.179
	DLP	0.03	0.31		0.03	0.04	
Vertical mismatch at implant base in mm	FFF	−0.48	0.54	−0.12	−0.49	−0.48	0.089
	DLP	−0.37	0.60		−0.37	−0.36	
Three-dimensional mismatch at implant tip in mm	FFF	1.43	0.64	0.49	1.42	1.43	**<0.001**
	DLP	0.93	0.45		0.93	0.94	
Mesio-distal mismatch at implant tip in mm	FFF	−0.51	0.69	−0.20	−0.51	−0.50	**0.003**
	DLP	−0.30	0.43		−0.31	−0.30	
Bucco-lingual mismatch at implant tip in mm	FFF	0.21	1.07	0.12	0.20	0.21	0.223
	DLP	0.08	0.56		0.08	0.09	
Vertical mismatch at implant tip in mm	FFF	−0.45	0.55	−0.11	−0.45	−0.45	0.121
	DLP	−0.35	0.61		−0.35	−0.34	

Statistically significant *p*-values are marked in bold font.

### Other factors

When examining the site of the implants, it was obvious that implants placed in the lower right quadrant showed a higher deviation from the virtually planned position compared to implants on the left lower quadrant. Except for the vertical mismatch at the implant base and tip the differences reached a statistically significance. The details are shown in Table 3. Considering the gender, the mean values of the female participants were lower compared to the mean values of the male participants thus, stating a higher accuracy for the female participants. The differences reached statistical significance for all parameters. The detailed data is shown in Table 4. When analyzing the handedness, age and a potential completed education prior to studying dentistry, only single parameters were different between the groups. No trend could be observed.

**Table 3:** Mean values and their standard deviations (SD), 95% confidence interval (CI) and *p*-values regarding the site of the implant.

Parameter	Implant site	Mean value	±SD	Mean difference	Lower limit 95% CI	Upper limit 95% CI	*p*-value
Three-dimensional angulation in degrees	Right	3.00	1.88	0.71	2.99	3.01	**0.001**
	Left	2.29	1.55		2.28	2.30	
Three-dimensional mismatch at implant base in mm	Right	0.91	0.46	0.13	0.90	0.91	**0.010**
	Left	0.77	0.40		0.77	0.78	
Mesio-distal mismatch at implant base in mm	Right	−0.28	0.37	−0.13	−0.28	−0.28	**0.001**
	Left	−0.15	0.30		−0.15	−0.15	
Bucco-lingual mismatch at implant base in mm	Right	0.25	0.46	0.36	0.25	0.25	**<0.001**
	Left	−0.11	0.39		−0.11	−0.11	
Vertical mismatch at implant base in mm	Right	−0.47	0.56	−0.08	−0.47	−0.46	0.212
	Left	−0.38	0.58		−0.39	−0.38	
Three-dimensional mismatch at implant tip in mm	Right	1.30	0.67	0.23	1.29	1.30	**0.001**
	Left	1.06	0.52		1.06	1.07	
Mesio-distal mismatch at implant tip in mm	Right	−0.49	0.62	−0.18	−0.50	−0.49	**0.010**
	Left	−0.32	0.53		−0.32	−0.31	
Bucco-lingual mismatch at implant tip in mm	Right	0.52	0.83	0.74	0.51	0.52	**<0.001**
	Left	−0.23	0.71		−0.23	−0.22	
Vertical mismatch at implant tip in mm	Right	−0.44	0.57	−0.08	−0.44	−0.44	0.223
	Left	−0.36	0.59		−0.36	−0.35	

Statistically significant *p*-values are marked in bold font.

**Table 4:** Mean values and their standard deviations (SD), 95% confidence interval (CI) and *p*-values regarding the gender.

Parameter	Gender	Mean value	±SD	Mean difference	Lower limit 95% CI	Upper limit 95% CI	*p*-value
Three-dimensional angulation in degrees	Female	2.43	1.48	0.92	2.42	2.44	**<0.001**
	Male	3.35	2.36		3.33	3.36	
Three-dimensional mismatch at implant base in mm	Female	0.79	0.41	0.23	0.78	0.79	**<0.001**
	Male	1.02	0.48		1.01	1.02	
Mesio-distal mismatch at implant base in mm	Female	−0.19	0.33	−0.10	−0.20	−0.19	**0.044**
	Male	−0.29	0.37		−0.29	−0.29	
Bucco-lingual mismatch at implant base in mm	Female	0.03	0.42	0.17	0.03	0.03	**0.010**
	Male	0.20	0.57		0.19	0.20	
Vertical mismatch at implant base in mm	Female	−0.36	0.57	−0.28	−0.37	−0.36	**0.001**
	Male	−0.64	0.53		−0.64	−0.63	
Three-dimensional mismatch at implant tip in mm	Female	1.09	0.53	0.37	1.09	1.10	**<0.001**
	Male	1.46	0.76		1.46	1.47	
Mesio-distal mismatch at implant tip in mm	Female	−0.36	0.57	−0.20	−0.36	−0.35	**0.012**
	Male	−0.56	0.61		−0.57	−0.56	
Bucco-lingual mismatch at implant tip in mm	Female	0.07	0.76	0.31	0.07	0.08	**0.011**
	Male	0.38	1.10		0.37	0.39	
Vertical mismatch at implant tip in mm	Female	−0.33	0.58	−0.28	−0.34	−0.33	**0.001**
	Male	−0.61	0.53		−0.62	−0.61	

Statistically significant *p*-values are marked in bold font.

### Required time for implant insertion

Overall, the mean time required for the insertion of the implants was 16:26 ± 5:52 min. For the implant insertion using the DLP guide, a mean time of 18:04 ± 5:19 min was recorded. For implant insertion using the FFF guide, the participants needed a mean time of 14:48 ± 5:57 min. The difference was statistically significant (*p* < 0.001).

### Evaluation of the questionnaires

The evaluation of the questionnaires revealed an exclusively theoretical knowledge in about 37.5% of the participants, while 12.6% of the participants stated to have theoretical and practical knowledge or only practical knowledge (10.5%). About one-third (31.3%) stated that they did not have neither theoretical nor practical knowledge about implant dentistry. When asked about their expectations prior to the hands-on course, the majority of the participants (83.3%) stated that they were fully met. Furthermore, it was indicated that the hands-on course stimulated the interest of the participants in implant dentistry. It could be shown that the attention to prosthetics and surgery regarding implant dentistry of the attendees was significantly increased. Considering the interests of the participants, the most urgent fields of interest were surgical techniques (43 participants) and implant planning (39 participants). Interestingly, the female participants revealed a statistically significant higher interest in implant planning (*p* = 0.043) and suturing techniques (*p* = 0.004).

## Discussion

### Comparison of transfer accuracy between guides made from DLP and FFF

When analyzing the accuracy of the really achieved compared to the planned implant position, the implants placed with the DLP guides showed a higher accuracy than the implants placed with the FFF guides. Due to the higher nozzle diameter of the FFF printer measuring 400 µm compared to 35 µm of the DLP printer, the strand diameter of the FFF guides is higher leading to a higher inaccuracy. This is in line with the current literature, where Abduo and Lau found a higher accuracy for the DLP printer [17]. In general, milled guides showed the highest accuracy, but have the disadvantage of a complex fabrication and, due to the subtractive nature of the process, tend to waste material [18]. The accuracy of surgical guides has to be considered as a multifactorial process where the accuracy of the guide is just one parameter [17]. One potential factor influencing the accuracy of the guide is the surface texture. For printed guides, it is depending on layer thickness, layer orientation and the post-processive shrinkage of the material [19]. In the present study, the participants rated the fitting of the guides on the respective models significantly different probably due to accuracy of printing technologies. The DLP strand diameter of 34 µm differs substantially compared to the nozzle size of about 400 µm when using the FFF technology. The differences in surface roughness will affect the fitting on the model and thus, might lead to a higher accuracy for the implant insertion using the DLP guide. Those findings are in line with a recent study, where a statistically significant difference was found between surgical guides made from DLP and FFF. The latter were consequently not considered as suitable for clinical application [7]. When comparing the results of the present study, they are comparable to the literature for the DLP printer where similar angular deviations and three-dimensional mismatches were reported [20, 21]. However, this is the situation in a clinical situation. When considering an educational situation in which the undergraduates should learn the principles of guided implant insertion and get familiar with the digital workflow, the lower accuracy of the FFF guides seems to be acceptable as the difference between DLP and FFF is in the deci-millimeter range. Additionally, both the initial and the running costs of the FFF workflow, are substantially lower compared to the DLP workflow. As it has to be kept in mind that dental schools are restricted in conducting hands-on courses mainly due to financial issues, this difference in costs does have an impact. Summarizing the facts above, the advantages in use the templates made by FFF are outweighing the disadvantages.

### Influence of other factors on the accuracy

Interestingly, the accuracy of transferring the implant position differed statistically significantly between the left and right mandible. A reasonable explanation could be the positioning of the participants on the right side of the phantom head. The soft tissue mask of the phantom head restricted the participants’ view of the implant site on the right mandible as well as complicating the access for the drill handle and surgical handpiece. On the left site, due to the absence of a tongue imitation, view and access to the implant site were possible without any major restricting structures. In contrary, Schnutenhaus et al. did not observe a statistically significant difference between the right and left implant site in a clinical study [22]. However, when analyzing the implant site, premolars and molars as well as upper and lower jaw sites were included. On the other hand, El Kholy et al. found in their study a statistically significant difference between the accuracy of implants placed in region 15 and 25 with surgical guides [23]. Interestingly, the accuracy was higher on the right site compared to the left site. One possible reason for this finding might be that the models were not mounted in phantom heads so that the models were placed on the laboratory workplace in the same position like mandibular models. Thus, the results of the mentioned study support our findings. The female participants showed a statistically significant higher accuracy compared to their male colleagues. This finding is in line with a study evaluating the didactic and psychomotor skills of dental students [24]. They observed better results for the psychomotor skills in females for certain preclinical practice tasks [24]. One possible reason for this might be that the male participants are not so diligent compared to female students and are more likely to overestimate their skills [25]. The findings of the present study have to be interpreted with caution with no equal number of female and male participants, potentially influencing the results. The other individual factors tested for a potential influence on the transfer accuracy showed no effect similar to previous laboratory studies in undergraduate dental students [15, 26, 27].

### Time required for implant insertion

When comparing the overall time for implant insertion without considering the mode, it is obvious that the participants needed more time in the current study compared to previous studies [15, 26, 27]. A reasonable explanation might be that during the current study the models were mounted in phantom heads with a soft tissue mask whereas in the studies mentioned above the models have been placed on the working table in front of the participants. Thus, factors hampering the visibility and access to the implant site, e.g. limited oral opening or the buccal tissue mask limiting the application of the drill-handle might extend the time required for the implant insertion. The difference between the guides might be explainable by the different fit assessed. In case of a tight fit, it might take longer to insert the drill-handle and drill as the guide is more rigid. A higher tolerance of the guide could suggest that the insertion of the surgical instruments might be less time consuming but resulting in a lower accuracy.

### Evaluation of the questionnaires

The fact that implant dentistry is an established therapy and should play a significant role in student education was not reflected by the answers of the questionnaire where about one third of the participants stated to neither have theoretical nor practical knowledge prior to the study. This is in line with the findings of a recent survey asking German undergraduates, educators and dentists about their knowledge and requirements for education in implant dentistry [10]. The results from the questionnaires underline the undergraduates’ request for an extended education in implant dentistry. Furthermore, undergraduates seem to benefit from hands-on courses, a fact having been shown in different studies [8, 11]. This should be considered when planning further student education. However, the hands-on courses are the most costly method of teaching potentially being limited in practicability due to financial restrictions [13]. In order to overcome this financial restriction, co-operation with industrial partners seems to be inevitable. An interesting finding when evaluating the questionnaires were the different interests stated by female and male participants. One possible reason might be that there is a general difference between female and male students regarding the specialties of dental medicine [28, 29]. Another potential reason might be that overestimation of the skills is more prominent in male students [25]. Thus, the male participants might assess that they already have sufficient skills in these areas. However, the assessment of the reasons was not topic of the present examination. As there was not an equal distribution of the gender and the cohort was relatively small, the results have to be interpreted with caution.

### Limitations

First, the study was carried out as a laboratory study using mandibular models. Thus, the accuracy might be higher compared to clinical studies due to the absence of factors influencing the surgeons work, e. g. saliva, blood, movement of the patient [30]. In the present study, it was tried to resemble a clinical situation by mounting the models into phantom heads with a soft tissue mask in order to restrict view and access to the implant site. A potential bias of the present study might be that the data was obtained in a voluntary course including participants more motivated compared to a mandatory course. However, Back et al. could show in their follow-up study, that the results gained from a voluntary cohort might be representative for the entire population of students [31]. Another limitation is that there might be a learning effect for the participants when inserting the second implant. In order to eliminate this potential factor, the guides and tooth position were set up alternating. However, the realization of an in vitro study allows a standardization of the experimental conditions and the elimination of potential influencing factors [23]. Furthermore, laboratory exercises can be advantageous in student education as a clinical situation can be simulated without exposing patients to a risk or burden [12, 32].

## Conclusion

In the present study, the hypothesis that the accuracy of fully guided implant insertion using guides made from DLP and FFF is comparable, has to be rejected. As the differences are in the deci-millimeter range and considering the initial and running costs, FFF guides seem to be suitable for the application in a laboratory hands-on course for the education of undergraduates. The influence of additional factors on accuracy has to be elucidated in further studies.

## Data Availability

The datasets supporting the conclusions of this article are available. Availability of data and materials by the corresponding author: Matthias.Schulz@med.uni-tuebingen.de.
